# Pro-Inflammatory Cytokine-Mediated Anemia: Regarding Molecular Mechanisms of Erythropoiesis

**DOI:** 10.1155/2009/405016

**Published:** 2010-03-01

**Authors:** F. Morceau, M. Dicato, M. Diederich

**Affiliations:** Laboratoire de Biologie Moléculaire et Cellulaire du Cancer, Fondation de Recherche Cancer et Sang, Hôpital Kirchberg, 9 Rue Edward Steichen, 2540 Luxembourg, Luxembourg

## Abstract

Anemia of cancer and chronic inflammatory diseases is a frequent complication affecting quality of life. For cancer patients it represents a particularly bad prognostic. Low level of erythropoietin is considered as one of the causes of anemia in these pathologies. The deficiency in erythropoietin production results from pro-inflammatory cytokines effect. However, few data is available concerning molecular mechanisms involved in cytokine-mediated anemia. Some recent publications have demonstrated the direct effect of pro-inflammatory cytokines on cell differentiation towards erythroid pathway, without erythropoietin defect. This suggested that pro-inflammatory cytokine-mediated signaling pathways affect erythropoietin activity. They could interfere with erythropoietin-mediated signaling pathways, inducing early apoptosis and perturbing the expression and regulation of specific transcription factors involved in the control of erythroid differentiation. In this review we summarize the effect of tumor necrosis factor (TNF)*α*, TNF-related apoptosis-inducing ligand (TRAIL), and interferon (IFN)-*γ* on erythropoiesis with a particular interest for molecular feature.

## 1. Introduction

Anemia represents a frequent complication in cancer patients as well as in chronic inflammatory diseases. It is an important cause of cancer-related fatigue [1], which considerably affects quality of life. Anemia is in fact considered as a bad prognostic factor for survival regardless of tumor type [2]. Up to 40% of cancer patients are anemic at diagnosis [3, 4] and the frequency even increases following chemotherapy [5]. This incidence varies according to the stage and the tumor type as well as patient age. Moreover, tumor responsiveness to radiotherapy seems to be weakened in the case of anemia [6]. In substitution to blood transfusion as anti-anemia therapy, some erythroid stimulating agents have been developed including human recombinant erythropoietin (hrEpo). Despite this treatment improves quality of life by alleviating anemia, the use of hrEpo as a treatment for cancer related anemia could be inappropriate for cancer patients. Indeed, based on clinical trials [7, 8] hrEpo was suspected to trigger tumor progression leading to decreased survival.

The essential role of circulating erythrocytes is the transport of oxygen to the tissues. Oxygen is bound to hemoglobin within erythrocytes that makes them highly prone to oxidative damage [9]. For this reason, erythroid cells contain numerous antioxidant enzymes to protect them against oxygen radicals [10] and deficient protection from reactive oxygen species (ROS) results in disease of red blood cells including anemia [11]. In fact, there are several causes of cancer-associated anemia including mechanical influence of the tumor on blood flow, and mainly the immune system activation with autoantibody formation and pro-inflammatory cytokines production [12]. Indeed, in vivo and in vitro studies have demonstrated the implication of interferon (IFN)-*γ*, tumour-necrosis factor (TNF)-*α*, TNF-related apoptosis-inducing ligand (TRAIL) and interleukin (IL)-1 [13–18] in the inhibition of the proliferation, and differentiation of erythroid progenitor cells. Moreover, anemia in children with solid tumours was related to IFN*γ* and TNF*α* [19]. In fact, pro-inflammatory cytokines were shown to trigger the suppression of renal erythropoietin production and therefore erythropoiesis. Inhibition of Epo production was shown in vitro and in vivo to potentially involve IFN*γ*, IL-1 and -6, and TNF*α* [20–22].

However, according to Spivak [12], the suppression of erythropoietin production in inflammatory conditions such as cancers, cannot be the solely explanation for anemia since the level of plasma erythropoietin is not affected in a sufficient amount. In this respect, hematopoietic stem/progenitor cells (HSPC) express receptors for pro-inflammatory cytokines [12] and several studies demonstrated that a direct action of the cytokines on hematopoietic cell lines in vitro could impair erythroid development and the number of erythroid progenitor cells [23–26]. Moreover, cytokines act in a microenvironment where they are produced and supposed to be concentrated, rather than in circulating blood. Indeed, poor correlation has been reported between circulating cytokine levels and the high cellular cytokine production [27]. Furthermore, marrow-adherent cells from patients with the anemia of chronic disease suppressed erythroid progenitors [28]. The molecular mechanisms involved in pro-inflammatory cytokine-mediated anemia, apart from Epo down-regulation and iron metabolism deficiency, are poorly described. For that reason, we aim to review the current knowledge concerning the direct effect of pro-inflammatory cytokines on erythroid cell differentiation, especially on signal transduction pathways and the regulation of erythrospecific genes expression in the pro-inflammatory-mediated inhibition of erythroid differentiation. We will then focus on molecular regulation of erythroid differentiation rather than on iron or erythropoietin involvement in anemia.

## 2. Molecular Regulation of Erythroid Differentiation

Expansion and differentiation of erythroid progenitor cells are dependent on growth factors and hormones network, acting in a thinly regulated chronology. Epo is the main erythropoietic hormone, acting by interaction with its specific membrane receptor EpoR. Stimulation of EpoR triggers the activation of signaling pathways required for survival, proliferation, and differentiation of erythroblasts. Another important cytokine involved in erythropoiesis is the stem cell factor (SCF), a ligand of the membrane receptor c-Kit. Signal transduction pathways activated by SCF have been reported to delay differentiation and to enhance progenitors proliferation in cooperation with Epo [29, 30].

EpoR is a homodimer constitutively associated with Janus tyrosine kinase 2 (Jak2). The activation of Jak2 results from the ligand binding-induced conformational change of the EpoR dimer [31, 32]. Activated Jak2 induces phosphorylation of the tyrosine kinase RON that activates PI3K *via* the docking molecule Grb2-associated binder (Gab)1 [33] also reported as phosphorylated after stimulation of EpoR [34]. Activation of the PI3K substrate AKT/PKB induces downregulation of the cell cycle inhibitor p27/kip1 expression [35] via inhibition of the transcription factor forkhead box 03a (FOXO3a) [36], a downstream target of EpoR/PI3K/AKT signaling pathway. Moreover, the transcription factor FOXO3a has been recently reported as one of the main regulators of oxidative stress in erythropoiesis [37]. In fact, FOXO3a is inactivated by Epo signaling pathway and its expression as well as its transcriptional activity is enhanced during the maturation of erythroid precursor cells when EpoR expression decreases. In the presence of Epo and in the case of the loss of FOXO3a, ROS mediate the decrease in lifespan of circulating erythrocytes as well as the rate of erythroid cell maturation. This suggested that FOXO3 is required for the regulation of oxidative stress in erythropoiesis. 

The cell-signaling cascade initiated by Epo-dependent Jak2 activation, leads to erythroblast expansion. Moreover, PI3K activation mediates the mitogen-activated protein kinase (MAPK, ERK1/2) path in correlation with the expansion of erythroblasts [38]. Another EpoR-mediated pathway leading to cell proliferation involves the Ras-Raf-MEK-ERK pathway [39–43] upon recruitment of the Grb2-Sos adapter molecules to the EpoR [44, 45]. Phosphorylation of the kinase Raf1 [39] has been shown to delay erythroblast differentiation by restraining the caspase-3 activation [46]. Moreover, EPO and SCF activate Jun-N terminal kinase (JNK) promoting proliferation and survival of hematopoietic cells [47, 48].

On the other hand, Epo-induced differentiation of erythroid cells is also dependent on PI3K/Akt signaling pathway that was suggested to act in concert with protein kinase C (PKC)-*α* [49]. PKC-*α* isoform has a role in mediating EPO-induced erythroid differentiation of the CD34+ progenitor cells from human bone marrow [50]. Furthermore, during Epo-dependent phase of erythroid differentiation, Epo and SCF suppressed activity of p38*α* whereas during the Epo-independent terminal-phase of differentiation, p38*α* and -*δ* phosphorylation was increased. This demonstrated both isoforms of p38 function to promote the late-stage differentiation of primary erythroid progenitors [51]. This confirmed previous report, showing that activation of p38 as well as JNKs was required for Epo-induced erythroid differentiation in SKT6 cells [52].

Also involved in erythropoiesis regulation is the Jak/STAT5 signaling pathway which is rapidly activated after Epo binding to EpoR, on erythroid progenitors. In mice models, it was shown that early erythroblasts survival as well as normal erythropoiesis was controlled by STAT5 [53]. Indeed, silenced STAT5 expression in mice led to an increase in early erythroblast numbers which nevertheless failed to progress in differentiation giving rise to anemia. Silenced STAT5-mediated anemia was correlated to down-regulation of the antiapoptotic bcl-X_L_ gene and to increased apoptosis. This supported the Jak/STAT5 pathway implication in the regulation of differentiation by preventing pro-erythroblasts apoptosis. On the other hand, bcl-X_L_-mediated inhibition of apoptosis in erythroid cells was shown to be a response to Epo/EpoR-induced inhibition of the caspase cascade amplification [54]. Indeed, caspase-3 activation led to degradation of the transcription factors SCL/TAL-1 as well as GATA-1, which regulate bcl-X_L_ gene expression [54, 55]. In fact, Tal-1 protein was shown phosphorylated in response to Epo stimulation [56] by PI3K-activated MAPK signaling pathway [57].

Stimulation of signaling pathways by Epo/EpoR, SCF/Kit or other stress conditions results in the activation/repression of many transcription factors specifically involved in erythropoiesis regulation. The zinc finger protein GATA-1 is considered as one of the most critical transcription factors in erythropoiesis as well as megakaryopoiesis. Besides GATA-1 that belongs to the GATA-family of transcription factors, GATA-2 is also involved in erythropoiesis and megakaryopoiesis regulation [58, 59]. Both GATA-1 and GATA-2 transactivation activities require interaction with friend of GATA (FOG)-1 cofactor [60, 61]. In addition, both transcription factors have GATA binding sites in their *cis*-acting elements allowing a cross-regulatory mechanism in which GATA-1 can control the expression of GATA-2 and vice versa. GATA-2 is overexpressed in early immature hematopoietic progenitors to ensure their maintenance and proliferation whereas GATA-1 is essential for the survival of erythroid progenitors as well as the terminal differentiation of erythroid cells [59, 62]. In fact, increased expression of GATA-2 determines megakaryocytic differentiation whereas its down-regulation is required for erythroid differentiation [58]. Recently, a role for GATA-2 in the regulation of quiescence in human hematopoietic stem and progenitor cells has been reported [63]. GATA-1 activation has been correlated to its phophorylation. Epo-induced phosphorylation of GATA-1 is important for maturation of fetal liver erythroid progenitor cells, specifically on serine 310 by PI3K/AKT that enhances GATA-1 transcriptional activity in vitro and in erythroid cells [64]. However, GATA-1 acetylation by CBP/p300 is also described as crucial for the binding to its DNA target GATA sequence possibly involving phosphorylation [65–67]. Moreover, phosphorylation of GATA-1 could be mediated by MAPK pathway, as an ubiquitination signal for its proteasomal degradation [68]. On the other hand, besides FOG1, GATA-1 activity is highly dependent on interaction with many cofactors including EKLF, SP1, CBP/p300, Lmo2, Ldb1, RUNX1, Fli1 and PU.1, which represent a part of the best-described interacting proteins. These cofactors can constitute a very complex network regulating erythropoiesis and megakaryopoiesis, by promoting or repressing GATA-1 activity [69–73]. Particularly, PU.1 is a strong inhibitor of GATA-1 DNA-binding activity and erythroid differentiation [74, 75].

## 3. TNF*α* Directly Inhibits Erythropoiesis

**Figure 1:**
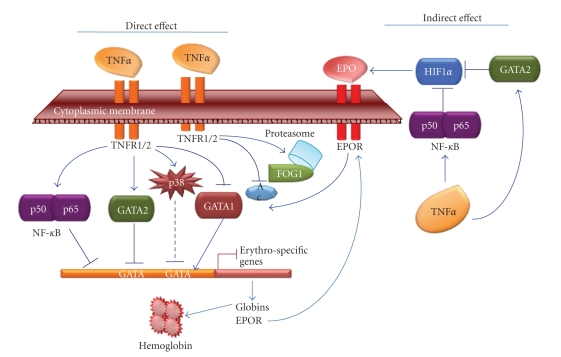
*TNF* *α* *inhibits erythropoiesis by direct and indirect effects*. In the indirect effect, TNF*α* activates the transcription factors NF-*κ*B and GATA-2, which were also reported as involved in Epo production inhibition by blocking HIF1*α* in vitro. Low level of Epo decreases the EpoR-mediated signaling pathways resulting among others, in the down-regulation of GATA-1, and consequently in a possible deregulation of EpoR expression. The direct effect of TNF*α* via its receptors TNFR1/2 has also been demonstrated. The activation of the NF-*κ*B canonical pathway (p50/p65) inhibits erythro-specific genes expression as globin genes. TNF*α* was also reported as activating GATA-2 whose over-expression is known to prohibit erythropoiesis in favor of megakaryopoiesis. Conversely, TNF*α* inhibits GATA-1 in K562, HEL and TF1 cells. GATA-1 expression is affected as well as its acetylation (Ac), and its interaction with FOG1 that was suggested to be degraded by proteasome. Moreover, TNF*α* was shown to rapidly stimulate p38MAPK phosphorylation in correlation with *γ*-globin gene down-expression while Epo had a delayed effect on this kinase activation. The combined effects of TNF*α* result in the decrease in erythro-specific genes expression and hemoglobin production.

Evidences for TNF*α* inhibiting effect on erythroid differentiation have been described 30 years ago. In 1987 Blick et al. observed a decrease in hemoblobin synthesis in cancer patients treated with TNF*α* (phase 1) [13] while in vitro study showed that TNF*α* inhibited the formation of BFU-E cells [28]. Later, Xiao et al. reported that TNF*α* inhibited the glycophorin A+ cells in correlation with an inhibition of erythropoiesis [76]. Moreover, an increasing hemoglobin level has been observed in patients suffering from anemia of chronic disease after an anti-TNF treatment [77]. The reduction of Epo production in the kidney partially explained the effect of the pro-inflammatory cytokins including TNF*α* [20]. La Ferla et al. reported that TNF*α*-mediated inhibition of Epo production in HepG2 cells was a consequence of GATA-2 and NF-*κ*B over-expression [78]. This was to some extent completed by Imagawa et al. who showed that TNF*α*-mediated inhibition of Epo gene expression could be rescued by the K-7174, a “GATA-specific inhibitor,” in HepG3 cells [79]. However, TNF*α* inhibiting effect on erythroid differentiation also occurs by a direct action on cells, including hematopoietic progenitors and cell lineages. Recently, Tsopra et al. published a study on disease-related anemia in chronic lymphocytic leukemia (CLL) patients. They showed that CLL-related anemia might result from the direct suppressive effect of TNF*α* on the erythroid development in early stages of erythropoiesis instead of an intrinsic defect of erythroid precursors to differentiate or to respond to Epo stimulation [80]. In fact, the results from Rusten and Jacobsen in 1995 suggested for the first time that TNF-*α*-induced inhibition of erythroid colony formation could be directly mediated on the progenitor cells [26]. By using BFU-E colony stimulated by various cytokine combinations (SCF, IL-3, IL-9) with Epo, they showed that TNF*α* inhibiting effect was mediated predominantly through p55-TNF receptor (TNFR1). This result was correlated to the implication of NF-*κ*B transcription factor, a TNF*α*/TNFR1 activated product, in the inhibition of erythroid specific genes. Especially, transfection assays in K562 cells showed the suppression of human *α*-like globin promoters by the NF-*κ*B pathway [81]**. **On the other hand, our group recently showed that TNF*α* inhibited hemoglobin production in aclacinomycin-induced K562 cells [25]. Aclacinomycin is an anthracyclin that was reported to induce over-expression of the key transcription factors for erythropoiesis, GATA-1 and NF-E2 in this cell line [82, 83]. Interestingly, the cytokine inhibiting effect was correlated to the down-regulation of GATA-1 and NF-E2. These results were confirmed in the erythroleukemia cell lines HEL and TF-1 [23, 24]. Furthermore, studies in K562 and HEL cells strongly suggested GATA-1 as a key target of TNF*α* inhibiting effect achievement (Figure 1). Indeed, the cytokine induced a decrease in the expression of FOG-1, an essential cofactor of GATA-1, a down-regulation of GATA-1 by proteasomal degradation and a reduced acetylation level of GATA-1 while the transcription factor GATA-2 was over-expressed [24]. In addition, an inhibition of EpoR, *α*- and *γ*-globin, erythroid-associated factor (ERAF), hydroxymethylbilane synthetase (HMBS), and glycophorin A (GPA) erythro-specific genes, was found in the Epo-dependent TF-1 cell line [23]. These results were concomitant with a reduction of GATA-1/FOG-1 complex formation and a significant and rapid increase in p38MAPK phosphorylation. The inhibition of p38 abrogated the inhibitory effect of TNF*α* on GATA-1 as well as *γ*-globin expression in Epo-induced TF-1 cells.

Thus, data related to TNF*α*-mediated inhibition of erythropoiesis show the indirect but also the direct involvement of this cytokine in anemia development. However despite few publications describe the molecular mechanisms implicated, they clearly show the role of TNFR1 and NF-*κ*B, as well as other specific transcription factors, namely GATA-1 and FOG1, NF-E2, and GATA-2. Notably, the down-regulation of GATA-1 at different levels by TNF*α* might affect erythroblasts programmed cell death besides differentiation, by triggering early apoptosis *via* down-expression of the anti-apoptic bcl-X_L_ gene whose transcription is regulated by GATA-1. Obviously, TNF*α* causes anemia independently of inhibition of Epo production. Further studies on molecular mechanisms using hematopoietic stem/progenitor cells should allow bettering understanding of how TNF*α*-mediated inhibition of erythropoiesis occurs.

## 4. TRAIL-Mediated Anemia Involves Apoptosis

TNF-related apoptosis-inducing ligand (TRAIL), also known as Apo2L, is a member of the TNF-related proteins initially identified and characterized by Wiley et al. in 1995. It is a type II membrane protein that is also expressed as a soluble protein. Both forms are able to induce apoptosis in a wide variety of transformed cell lines of diverse origins [84] including several hematopoietic lineages [85]. TRAIL exhibits structural and functional similarities with Fas ligand (FasL/CD95L), including the use of FADD as adaptor molecule [86–90]. It interacts with four high-affinity membrane receptors (R) TRAIL-R1 (DR4), TRAIL-R2 (DR5), TRAIL-R3 (DcR1) and TRAIL-R4 (DcR2) that belong to the apoptosis inducing TNF-receptor family. A study on TRAIL implication in the homeostatic control of hematopoiesis showed its negative effect on normal erythropoiesis, in a differentiation-stage specific manner [18].

Moreover, studies on TRAIL activity and expression in myelodysplastic syndromes (MDS) patients have been reported. MDS are characterized by impaired erythropoiesis leading to anemia, the major clinical feature in this syndrome. By analyzing MDS marrow, Zang et al. showed that TRAIL induced extensive apoptosis, including in the blast cell population while no increase in apoptosis was observed in normal marrow [91]. They correlated this observation with high levels of surface expression of agonistic receptors TRAIL-R1 and -R2 in MDS marrow, which could explain the selective killing of tumor cells by TRAIL. On the other hand, the authors suggested that apoptotic response could be modified by the variations in the anti-apoptotic protein FLIP expression in addition to the cell-surface-receptor expression in MDS. Indeed, they found that FLIP was expressed in marrow from healthy donors whereas the protein was not detectable in most of the MDS marrows. Together, the results suggested that TRAIL might play a role in the regulation of hematopoiesis in MDS marrow. In the same way, another study showed that TRAIL expression was increased in the bone marrow mononuclear cells from MDS patients and released at the bone marrow level probably contributing to the degree of anemia. In fact, MDS bone marrow-conditionned media with released soluble TRAIL, added to a normal CD34-derived erythroblasts culture, led to impairment of erythroid maturation, as assessed by the levels of GPA. This demonstrated the role of soluble TRAIL in affecting the maturation of erythroid precursors in MDS patients [92].

A comparative study in multiple myeloma (MM) patients with or without anemia, showed an inverse correlation between the expression of TRAIL (and Fas-L) in malignant plasma cells and the relative erythroblast numbers, with a higher percentage of immature erythroblasts in enriched erythroblast populations from anemic MM patients [93]. A downexpression of GATA-1 transcription factor has been detected in immature erythroblasts from MM patients with severe anemia. They ascribed this decrease to Fas-L and/or TRAIL-mediated cleavage of GATA-1 native form. Indeed, GATA-1 can be cleaved by several caspases in CD34+ cell-derived erythroblasts, following their treatment with TRAIL leading to maturative arrest [55].

Secchierro et al. found that TRAIL-R3 and -R4 were never expressed neither on the surface of freshly purified CD34+ hematopoietic progenitor cells nor on GPA+ erythroblasts at early-intermediate and late culture times [94]. On the contrary, they suggested that TRAIL-R2 probably played a role in erythroid development since this receptor was found expressed at early phases of erythroid differentiation with a progressive increase along the erythroid maturation. Conversely, TRAIL-R1 was detectable only at early phases of erythroid development. The authors reported that TRAIL induced MAP kinase ERK1/2 activation in primary normal erythroblasts, but not p38 or JNK. Considering that ERK1/2 pathway was shown as involved in the early proliferative phases of erythropoiesis [95] and in the inhibition of terminal erythroid differentiation [52, 96–98], they suggested that TRAIL-mediated activation of ERK1/2 was responsible for the inhibiting effect of TRAIL on normal erythroid development. On the other hand, the activation of ERK1/2 by TRAIL at all the stages of erythroid development correlated with the expression of TRAIL-R2. Together these studies clearly indicate that TRAIL plays a role in erythropoiesis inhibition and anemia development *via *its receptor R2 and probably R1.

## 5. Indirect Action of Interferon-Gamma

Interferon (IFN)-*γ* has been reported as inhibiting growth and differentiation of erythroid precursor cells [99, 100] as well as a potential mediator of hematopoietic failure in aplastic anemia (AA). AA belongs to the bone marrow failure syndromes, characterized by a breakdown in the stem- and progenitor-cell compartments. In fact, several authors had suspected the role of IFN-*γ* in erythropoiesis inhibition according to an elevated production of the cytokine by lymphocytes in AA patients while it was absent in normal bone marrow [100–105]. A microarray analysis of RNA-expression profile in CD34+ and bone marrow stroma cells showed that IFN-*γ* induced an increase in the expression of Fas and TRAIL genes, known to be involved in anemia promotion [106]. Furthermore, Felli et al. reported that the TNF family members TRAIL and TWEAK (Apo-3 ligand) as well as its receptor Fn-14 were involved in IFN-*γ*-mediated suppression of erythropoiesis in purified human erythroblasts [107]. This was in correlation with the inhibition of growth and differentiation of the erythroblasts. Moreover, they could restore erythroid cell survival, proliferation and maturation, inhibited by IFN-*γ*, performing combined neutralization of TWEAK, TRAIL and FasL/CD95L. This pointed out the role of the three proteins as effectors of IFN-*γ* in erythropoiesis suppression. On the other hand, the modulation of adhesion molecule genes expression (ICAM1 and VCAM1, integrin-*α*5 and integrin-*β*3) in stromal cells treated with IFN-*γ* [106] correlated with previous works demonstrating the altering effect of IFN-*γ* (and TNF-*α*) on the adhesive mechanism [108]. Therefore it was assumed that the cytokine may affect the function of bone hematopoietic stem cells by disturbing their adhesion to the marrow microenvironment in AA patients [106]. Until now, in opposition to TNF*α* and TRAIL, the direct effect of IFN-*γ* on erythroblastic cells at the molecular level has not been demonstrated. However, IFN-*γ* and also IL-6 are known to activate the phosphorylation of the transcription factor STAT3. It was recently reported that STAT3 activation silenced *γ*-globin gene expression in primary erythroid cells [109] and foetal hemoglobin production in K562 cells. In these experiments the effect of STAT3 was reversed by GATA-1 induced over-expression [110].

## 6. Inflammation, Oxidative Stress and Anemia

One of the major proteins of red blood cells is the acidic peroxidoxin, which plays a role in the response against oxidative stress. The expression of this protein is induced early in erythropoiesis, prior to hemoglobin production suggesting the importance of the protection of erythroid progenitors against oxidative stress [111]. Erythropoiesis regulation is indeed very sensitive to oxidative stress and the release of proinflammatory cytokines in neoplastic patients as well as chronic inflammatory diseases are often associated with increased production of reactive oxygen species (ROS) (H_2_O_2_ and HO^∙^) [112, 113]. Moreover, TNF*α* links inflammation to carcinogenesis through ROS [114] and it was reported that TNF*α* elevates ROS production in glioma cells [115]. However, it appears that the role of oxidative stress is complex since reactive oxygen species may either trigger [116] or prevent [117, 118] hematopoietic differentiation and proliferation. It was demonstrated that the increase in oxidative stress and free radicals are associated with disorders implicated in anemia of chronic disease [119] while anthracycline- and butyrate-mediated differentiation of K562 cells was prevented by anti-oxidant compounds [120]. This last study suggested that butyrate- or anthracycline-generated oxidative stress was involved as the first step in the irreversible erythroid differentiation process. On the other hand, Dallalio et al. reported that inflammatory cytokine-mediated anemia of chronic disease may occur through modulation of oxidative stress [121]. Elorza et al. also demonstrated the importance of the ROS modulation during erythropoiesis, by studying the uncoupling protein (UCP)2 role [122]. Its expression leads to a decrease in mitochondrial superoxide and it was previously shown that UCP2 was related to erythroid differentiation since it was induced by GATA-1 activation in the proerythroblast G1E-ER cell line [123]. UCP2 deficiency in progenitor cells at the Epo-dependent phase of erythropoiesis led to a decrease in cell proliferation in correlation with ERK reduced phosphorylation, which is known as a ROS-dependent cytosolic regulator of cell proliferation. A relationship between UCP2 and the MAPK/ERK pathway has been reported in the case of elevated inflammatory response and inhibition of UCP2 could lead to the development of anemia [124].

## 7. Conclusions

Obviously pro-inflammatory cytokines have a negative effect on erythropoiesis development leading to anemia in multiple diseases including chronic infections, chronic inflammatory diseases, myelodysplastic syndromes, and malignancy. The pathophysiological effects of cytokine over-expressions have been well described but the mechanisms surrounding cytokine-mediated induction of anemia remain largely unknown. Many studies show that erythroid colony formation in response to Epo is impaired in the presence of pro-inflammatory cytokines [125–127] without providing molecular mechanisms. In this paper we review data available on the molecular mechanisms involved in the defect of erythropoiesis due to three pro-inflammatory cytokines TNF*α*, TRAIL, and IFN*γ*. Nonetheless, other pro-inflammatory cytokines have been involved in erythropoiesis defect, such as the interleukin (IL)-6 [112]. Impaired erythropoiesis is most likely due to apoptosis induction, cell growth inhibition and EpoR downregulation as a result of a local increased production of the cytokines, but also iron metabolism damage. In all cases pro-inflammatory cytokines affect Epo either by inducing inhibition of its production by kidney or by preventing its physiological functions at the cellular level. Indeed, cytokines activate signaling pathways that can have common feature with EpoR-triggered signaling pathways leading to cell proliferation, differentiation, or survival.

Regulation of erythropoiesis occurs in a tightly time dependent manner and changes in the timing of one specific signaling intermediate activation can disturb cell differentiation process. Thus, it is known that both TNF*α* and Epo activate p38MAPK phosphorylation. We could recently observe that this kinase was very early activated by TNF*α* (10 minutes) while Epo-mediated activation occurred much later in TF-1 cells (48 hours). This result was correlated to the inhibiting effect of TNF*α* on Epo-induced erythroid differentiation in TF-1 cells [23]. Thus, how specific signal transduction pathways of pro-inflammatory cytokines can interact with EpoR ones to prevent erythroid differentiation is an interesting issue. On the other hand, pro-inflammatory cytokines activate diverse transcription factors that can have contradictory effects in regard to the cell intention. For example, NF-*κ*B, which is typically activated by cytokines, was reported involved in the inhibition of Epo production [78] and the repression of globin genes expression [81]. Moreover, GATA-1/GATA-2 balance, which is a key element for erythropoiesis regulation was shown affected by TNF*α* [24]. 

On the other hand, besides erythroid genes expression, GATA-1 and TAL-1/SCL are implicated in apoptosis regulation by controlling bcl-X_L_ gene expression. Common pro-apoptotic properties of pro-anemia cytokines seem involved in erythropoiesis delay or prevention. Therefore, cytokine-mediated activation of caspases and Epo-induced GATA-1 expression might represent a crossing point in which GATA-1 cleavage would lead to down-regulation of Bcl-Xl expression and then apoptosis activation. Studying the pro-inflammatory cytokines effect at the transcriptional level should allow understanding how the erythroid specific genes are repressed and how the pro-apoptotic genes are activated. In addition to transcription factors network analysis, especially GATA-1, micro-RNAs implication could be taken in consideration according to the expected role of these molecules in erythropoiesis [128–135].

In conclusion, the modulation of Epo-mediated signaling pathways as well as transcription factors and cofactors by pro-inflammatory cytokines are required to achieve inhibition of erythroid differentiation. Several in vitro and in vivo studies demonstrated that high levels of proinflammatory cytokines and increased oxidative stress contribute both to the development of anemia and to the resistance to recombinant human hrEpo. The complexity of this phenomenon provides multiple targets for potential drugs in order to inhibit cytokines effect and/or to promote erythroid differentiation, mainly in cancer-related anemia. Indeed, despite hrEpo is clinically efficient for anemia treatment it is strongly suspected to induce tumor cell proliferation. Therefore studies of the mechanisms involved in the inhibiting effect of cytokines on erythropoiesis are essential to intend future anti-anemic treatment, at least for cancer patients.
